# Racial Variations in Emergency Department Management of Chest Pain in a Community-based Setting

**DOI:** 10.51894/001c.32582

**Published:** 2022-02-24

**Authors:** Elisabeth Greenberg, Elle Schultz, Emily Cobb, Shelia Philpott, Megan Schrader, Jessi Parker

**Affiliations:** 1 Resident Physician Spectrum Health Lakeland Emergency Medicine Residency; 2 Core Faculty Spectrum Health Lakeland Emergency Medicine Residency; 3 Biostatistician Lead Spectrum Health

**Keywords:** Chest Pain, Racial Disparities, Emergency Medicine, Race

## Abstract

**INTRODUCTION:**

Chest pain is one of the most common chief presenting complaints occurring in most Emergency Departments. The HEART score is a validated risk stratification tool commonly used to evaluate chest pain. Prior research has demonstrated the existence of complex racial variations in health care, specifically in what tests are ordered (or accepted by patients) during evaluation and treatment of cardiac disease. The authors hypothesized that chest pain management (i.e., disposition to hospital/observation unit and rates of stress testing) patterns and longitudinal outcomes (i.e., death and 30-day readmission) would occur differently in African Americans despite systematic use of the HEART score.

**METHODS:**

Funded by the Statewide Campus System, this study was comprised of a retrospective chart review of a sample of eligible patients presenting with chest pain to the authors’ 345-bed community-based Michigan hospital.

**RESULTS:**

Of the 1,412 eligible sample patients, 886 (63%) reported their racial affiliation as White, 473 (33%) African-American, and 53 (4%) “Other”. The average HEART score in Whites was 3.92 (SD = 1.89) compared to 3.31 (SD = 1.79) in African-Americans, (p < 0.01, 95% CI: 0.40-0.82). However, White patients’ odds of admission to observation or inpatient was 1.49 times higher (95% CI: 1.04 - 2.15), with every unit increase in HEART score increasing the odds ratio of admission by 3.24 times (95% CI: 2.79 - 3.76). White patients were also 2.37 times more likely to receive (or accept) stress tests than African American patients (95% CI: 1.41 - 3.88). Only five (0.01%) of 458 White patients with HEART score between 4 and 6 experienced 30-day readmission or death whereas seven (0.04%) of 193 African-American patients experienced these outcomes (p = 0.04 with OR 3.40, 95% CI: 1.07 - 10.9).

**CONCLUSIONS:**

Although the authors were unable to precisely distinguish the provider (e.g., desire to order testing) and patient-driven (e.g., desire to accept testing) factors likely to contribute to measured differences, these results suggest continued complex racial variations concerning hospital admission and stress testing in chest pain patients. Further studies are needed to analyze potential systems or subject-level factors influencing the multi-dimensional phenomenon of chest pain management across racial affiliation.

## INTRODUCTION

Growing medical research has demonstrated the ongoing existence of variations in the ordering and reception of cardiac health care services. Racial differences have been shown for cardiac diagnoses across numerous settings, including patients with suspected Acute Coronary Syndrome (i.e., “heart attack”).^1–4^ More specifically, African Americans are historically less likely to undergo (or accept) stress testing,^1,2^ be hospitalized for coronary syndromes ^5^ and be referred for a cardiac catheterization ^3,6^ when presenting to Emergency Departments (ED) with a complaint of chest pain.

Chest pain is one of the most common ED complaints, with over six million annual visits.^7^ Those diagnoses associated with chest pain complaints range from minor, self-limited ailments (e.g., acid reflux, viral illnesses) to acute life-threatening diseases (e.g., heart attack or blood clot). Nearly one in four Americans die from heart disease each year^8^ with minorities such as African-Americans being twice as likely to die of heart disease in comparison to other minority groups.^9^ Differentiating patients with cardiac ischemia from those with more benign causes of chest pain is a primary management concern of ED physicians.^5^

Many ED units have developed protocols and specialized diagnostic units for the management of chest pain.^10^ These practice developments have been associated with improvements in health outcomes (e.g., a reduction of in-hospital stroke, vascular complications, bleeding, transfusion, and death).^10^ Risk stratification is a key aspect of protocolized chest pain care.^11^ Many ED now rely on the validated HEART Pathway to risk stratify subjects presenting with chest pain. The HEART score method includes measurement of a patient’s History, ECG changes, Age, Other Risk Factors (e.g., hypertension, obesity, smoking history, etc.), and Troponin value to place a patient into “low risk”, “moderate risk” or “high risk” categories for a major cardiac event.^11^

During two 2010 and 2013 HEART score validation studies, there was a predominance of White sample subjects.^11,12^ Follow up research for the HEART score has demonstrated effective chest pain risk stratification for different ethnic and racial subgroups.^13,14^ However, there have been limited community-based studies examining whether HEART score utilization can be used to investigate racial differences in chest pain management.

### Purpose of Study

The purpose of this study was to evaluate the relative influence of HEART scores on the management of African-American patients presenting to a community-based ED with chest pain. The clinician authors (i.e., all but last author JP) had hypothesized that management (i.e., disposition to hospital/observation unit and rates of stress testing) and longitudinal outcomes (i.e., death and 30-day readmission) variations would be significantly different in African-Americans compared to White patients despite systematic use of the same HEART score protocol.^11^

## METHODS

### Study Design

Before data collection, this study design was approved as an IRB Exempt project. The study population included adult patients presenting to the selected ED with chest pain from 8/14/2018 through 9/30/2019. The authors’ Michigan-based hospital has an annual ED volume of 78,280 visits with 345 hospital beds. This study window coincided with an update to the electronic health record (EHR) in which the HEART score tool was auto populated into provider notes for patients with a chief complaint of chest pain. Each of the five components of the HEART score were entered by the provider and then the tool software calculated the final risk score.

Descriptive scoring guides and risk definitions from the selected HEART protocol were included in the tool. A score of between 0 - 3 was considered “low risk”, with 0.9 - 1.7% risk of cardiac event, indicating the patient could be safely discharged home.^11,12^ A score of between 4 - 6 was considered “moderate risk”, with a 12 - 16.6% risk of cardiac event, indicating that the patient could be either observed in the hospital or discharged home. A score of 7 or greater was considered “high risk”, with a 50 - 65% risk of cardiac event, indicating the patient should be considered for early intervention evaluation. Final management and disposition were left to the discretion of the treating provider after speaking with the patient regarding their condition and what healthcare services they were receptive to.

This study targeted patients greater than 40 years old, who presented for evaluation of chest pain, with evidence of cardiac workup defined as a troponin lab value and EKG, and a documented HEART score. A focus was placed on patients older than 40 years old to align with earlier HEART Score validation studies (i.e., whose average participant age was 61 years old +/- 15 years).^6,7,12^

Data was retrieved from the authors’ Epic, version August 2019 EHR via a Microsoft SQ server. (SQL server tools and code available upon request). The data set included all eligible patients’ socio-demographic characteristics (i.e., Age, Gender, Racial Affiliation) and information regarding their ED workup. The server tool was also used to extract subsequent information including index hospital admission, provocative cardiac testing (e.g., stress test), as well as death and 30-day readmissions.

Other information concerning whether the patients received a chest x-ray, Cardiology consultations, and/or EKG and risk factors including Tobacco Use status (i.e., never, former, or current), or documented history of Stroke, Hypertension and Diabetes were also extracted. Data were then entered into a spreadsheet for validation and chart abstraction, which was performed by our research group to confirm the overall reliability of the extraction tool. All data was stored in a de-identified manner on password protected computers accessed only by the authors.

For this study, “Management evaluation” was primarily defined by whether the admitted patient received a stress test.^11^ The HEART score does not explicitly suggest management evaluation but stress tests are a common modality in the work up of chest pain.^2,12,13^ Similar to earlier HEART score studies, “Disposition” was defined as being discharged home or admitted to the hospital - either inpatient or being placed for observation in the hospital Chest Pain Unit (CPU).^1,11^

### Study Outcomes and Analyses

The primary outcomes of interest included patient management and disposition patterns after their ED encounter. All analyses were executed by last author JP using SAS (SAS Enterprise Guide software, Version 7.1, SAS Institute Inc, Cary, NC). Baseline characteristics of study patients, including their HEART scores, were compared between African-American and White patients. A series of univariate analyses (Chi-Square or Two Sample T Test as appropriate) were performed on the variables Age, Gender, Racial Affiliation, Tobacco Use, Hypertension, Diabetes, history of Stroke and HEART score to look at possible contributors to admission or stress test utilization.

HEART scores were then stratified by low risk (i.e., score 0 - 3), medium risk (i.e., score 4 - 6) and high risk (i.e., score > 7). Racial Affiliation and Gender were specifically analyzed via Cochran-Mantel-Haenszel (CMH) tests to examine whether admission rates were different while accounting for HEART score risk groups.

Finally, a series of multivariate logistic regression models were conducted to investigate which of the selected variables independently predicted whether each patient was admitted. This analytic procedure was repeated to compare CPU patients who had received a stress test versus those who had not. Based on the univariate analyses results, if the p-value was less than 0.10 they were retained in the developing stepwise model using backwards selection to for consideration in the final model observing a statistically significant p value of 0.05 to indicate statistical significance.

Initially, 974 (70%) of sample records were randomly selected to build the model. The remaining 418 (30%) of study records were used to validate the model. Odds ratios with 95% confidence limits were produced for the variables included in the final model.

## RESULTS

### Admission to Hospital or CPU Observation Status

A systematic EHR extraction yielded a total of 1,412 patients during the study window. The average mean sample age was 60.4 (SD = 15.7) years for White subjects, 53.4 (SD = 15.7) for African-American subjects, and 52.4 (SD = 16.2) for “Other”. Men comprised 659 (46.7%) of the data set with most of the sample being White, 887 (62.8%). The “Other” category (n = 53) (i.e., Hispanic, Asian, and Native Americans) was excluded from later analysis due to an inadequate level of subgroup statistical power. Subject socio-demographics, cardiac risk factors, and HEART scores are presented in Table 1. As can be seen in the table, African-American patients were significantly younger, more likely to be female, had higher rates of Hypertension, Tobacco Use, and had lower HEART scores.

**Table 1.** Socio-Demographic and Clinical Characteristics, HEART Scores by Race

**Variable**	**Caucasian (N = 886)**	**African Americans (N = 473)**	Other* (N = 53)	**p-value**
**Age** (mean, y)	60.4 (SD = 15.7)	53.4 (SD = 15.7)	52.4 (SD = 16.2)	**< 0.01**
**Gender** (% Male)	49.9%	40.8%	45.3%	**0.01**
**Tobacco** Use (%)	59.3%	63.2%	35.9%	**< 0.01**
**Hypertension** (%)	48.8%	53.7%	32.1%	**< 0.01**
**Diabetes** (%)	11.2%	13.5%	11.3%	0.44
				
**HEART Score Groups** (%)				
Low (0-3)	353 (39.7%)	261 (55.2%)	32 (60.4%)	**< 0.01**
Moderate/High: (4+)	534 (60.3%)	212 (44.8%)	21 (39.6%)	

*Other includes Hispanic, Asian, and Native American

As depicted in Table 2, the average HEART score was significantly higher in White patients (Mean 3.92, SD = 1.89) than African-Americans (mean 3.31, SD = 1.79) (p < 0.01). There were also significant differences between chest pain patients admitted onto a hospital unit or CPU observation status (Table 2).

**Table 2.** Socio-demographic and Clinical Characteristics of Hospital-admitted vs. Non-admitted Patients

**Variable**	**Not Admitted (N = 633)**	**Admitted (N = 779)**	**p-value**
**Age**	50.2 (SD = 14.0)	63.8 (SD = 13.4)	**< 0.01**
**Gender** (Male)	252 (39.8)	407 (52.2)	**< 0.01**
**Race**			
White	344 (54.3)	542 (69.6)	
African-American	259 (40.9)	214 (27.5)	**< 0.01**
Other *	30 (4.7)	23 (3.0)	
**Tobacco Use**	N = 625	N = 777	
Never Smoker	281 (45.0)	284 (36.6)	
Former Smoker	156 (25.0)	297 (38.2)	**< 0.01**
Current Smoker	188 (30.1)	196 (25.2)	
**Hypertension**	258 (40.8)	445 (57.1)	**< 0.01**
**Diabetes**	54 (8.5)	115 (14.8)	**< 0.01**
**History of Heart Attack**	0 (0.0)	8 (1.0)	**0.01**
**History of Stroke**	34 (5.4)	82 (10.5)	**< 0.01**
**HEART Score**	2 ± 1	5 ± 2	
Low: 0-3	538 (85.0)	107 (13.7)	**< 0.01**
Medium: 4-6	92 (14.5)	579 (74.3)	
High: 7+	3 (0.5)	93 (11.9)	

*Other includes Hispanic, Asian, and Native American

Cochran-Mantel-Haenszel procedures were used to examine the potential significance of Gender and racial affiliation while controlling for HEART score category. As depicted in Table 3, White patients were significantly more likely to be admitted to the hospital or CPU than their African American counterparts (low risk, 19.3% versus 13.4%; moderate/high risk, 88.8% versus 84.4%; p-value = 0.01) when accounting for HEART score category. Men were also more likely to be admitted or observed than women (low risk, 20.2% versus 14.2%; moderate/high risk 88.3% versus 86.9%) but this variation was not statistically significant (p = 0.06).

**Table 3.** Hospital Admissions and Stress Tests based on Racial Affiliation

	**Whites**	**African- Americans**	**p-value**
**Hospital Admissions**			
Low risk%	19.3% (68, N = 352)	13.4% (35, N = 261)	**0.01**
Moderate/High risk %	88.8% (474, N = 534)	84.4% (179, N = 212)	
**Stress Tests**			
Low Risk%	5.4% (19, N = 352)	0.8% (2, N = 261)	**< 0.01**
Moderate/High Risk%	20.2% (108, N = 534)	13.7% (29, N = 212)	

* Cochran-Mantel-Haenszel testing

**Figure 1.**
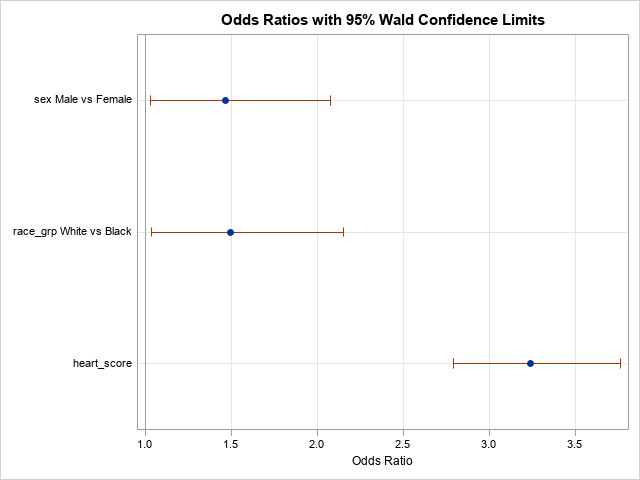
Factors Influencing Hospital Admission Rates

The final multivariate logistic regression model was used to analyze the influence of history of Hypertension, Tobacco Use, Diabetes, Age, Gender, Racial Affiliation, and HEART score upon potential hospital admission. Only Gender, Racial Affiliation, and HEART score were statistically significant contributors (Figure 1). Males had an odds ratio of 1.47 greater than women of being admitted (95% CI: 1.03 - 2.08), Whites had an odds ratio of 1.50 times increased likelihood of admission (95% CI: 1.04 - 2.15), and every unit increase in HEART score increased odds ratio of their admission by 3.24 times (95% CI: 2.80 - 3.76).

There were also several significant differences between the characteristics of those sample patients who received a stress test and those who did not (Table 4).

**Table 4.** Comparison of Patients receiving Stress Testing vs. Others

**Variable**	**No Stress Test (N = 1250)**	**Stress Test (N = 162)**	**p-value**
**Age**	57.2 (SD = 15.6)	61.7 (SD = 11.5)	**< 0.01**
**Gender** (Male)	569 (45.5%)	90 (55.6%)	**0.02**
**Racial affiliation**			
White	759 (60.7%)	127 (78.4%)	**< 0.01**
African-American	442 (35.4%)	31 (19.1%)	
Other *	49 (3.9%)	4 (2.5%)	
**Tobacco Use**	N = 1241	N = 161	
Never Smoker	504 (40.6%)	61 (37.9%)	0.66
Former Smoker	396 (31.9%)	57 (35.4%)	
Current Smoker	341 (27.5%)	43 (26.7%)	
**Hypertension**	616 (49.3%)	87 (53.7%)	0.29
**Diabetes**	143 (11.4%)	26 (16.0%)	0.09
**History of Heart Attack**	5 (0.4%)	3 (1.9%)	0.05**
**History of Stroke**	102 (8.2%)	14 (8.6%)	0.83
**HEART Score**	4 (SD = 2)	5 (SD = 1)	**< 0.01**
Low: 0-3	623 (49.8%)	22 (13.6%)	
Medium: 4-6	542 (43.4%)	129 (79.6%)	
High: 7+	85 (6.8%)	11 (6.8%)	

*Other includes Hispanic, Asian, and Native American

**Cochran-Mantel-Haenszel testing

**Figure 2.**
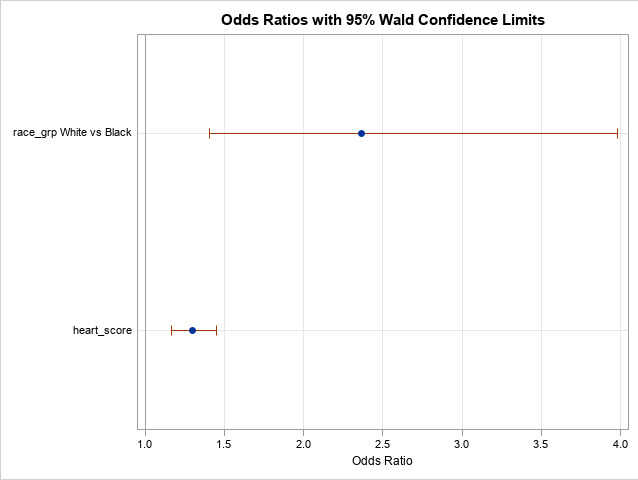
Factors influencing Hospital Admission and Stress Testing

A multivariate logistic regression model was created to evaluate for which factors independently predicted hospital admission and stress testing. Based on the results depicted in Tables 3 and 4, Gender, Racial Affiliation, Age, and HEART score category terms were included in the model (Figure 2). For stress testing, only Racial Affiliation and HEART score category contributed independently.

### Longitudinal Outcomes

Thirty-day outcome results are reported in Table 5. Those patients who were admitted to the CPU or the same/another hospital unit (n = 17, 2.2%) were significantly more likely to be readmitted within 30 days than those who were not originally admitted (0.2%; N=1) (p < 0.01). As can be seen in this table, the outcome frequencies of 30-day deaths for sample subgroups were quite low and non-significant.

**Table 5.** 30-day Hospital Readmissions and Mortality for Total Sample Patients

**Variable**	**Not Admitted (N = 633)**	**Admitted (N = 779)**	**p-value**	**No Stress Test (N = 1250)**	**Stress Test (N = 162)**	**p-value**
**Readmission 30 Days**	1 (0.2%)	17 (2.2%)	**< 0.01**	17 (1.4)	1 (0.6)	0.71*
**Ed Visits with 30 days**	99 (15.6)	126 (16.2)	0.78	203 (16.2)	22 (13.6)	0.38
**Death 30 days**	0 (0.0)	3 (0.4)	0.26*	2 (0.2)	1 (0.6)	0.31*

*Fishers Exact Test utilized; all other categories evaluated via Chi-Squared Test.

## DISCUSSION

The HEART Score has been used as an objective tool to standardize chest pain treatment decisions and is widely accepted in Emergency Medicine settings.^11,12^ Using this measure, our results demonstrate likely-complex racial variations in score-controlled rates of hospital admission and stress testing. Similar to earlier studies,^2,9^ we observed increased morbidity and mortality levels in African American patients assigned different HEART risk scores that reached significance (Table 5). Other studies with similar designs have also demonstrated lower relative chest pain management patterns,^13^ decreased rates of admission, invasive testing for chest pain ^14^ and decreased rates of percutaneous intervention (heart catheterization) in African American sample subroups.^15^

Other measures such as the Thrombolysis in Myocardial Infarction (TIMI) score method have been used to investigate this complex ED care phenomenon.^2^ However, the HEART score method has been shown to better discriminate patients possessing lower-risks than the TIMI or other measures for major adverse cardiac events.^16–20^ It has been earlier acknowledged that such standardized score methods could actually introduce potential biases on the final results of study groups.^21,22^ It will also remain quite difficult for researchers to precisely tease out what chest pain management patterns may be primarily attributable to variations in provider ordering, patient preferences, or other unmeasured factors.^3,9,23^

Readers should still consider that such standardized chest pain protocols have been shown to decrease racial treatment variations, reduce long term healthcare costs and improve care access.^10,24,25^ For example, at our Michigan-based ED setting, we are creating our own clinical care protocol to standardize ordering of cardiac imaging on our CPU patients to help decrease management variations based on individual patient HEART scores.

### Study Limitations

First, our analysis did not evaluate unmeasured factors (e.g., number of prior ED visits, patient preferences, timing of earlier stress tests, established cardiac clinic follow ups, ED census at time of encounter) that could have played a role in patient management outcomes. In addition, we were unable to capture the number of sample patients who may have obtained a clinic-based stress test shortly after discharge. Finally, we experienced difficulties obtaining complete retrospective longitudinal EHR documentation concerning 30-day follow-up data.

## CONCLUSIONS

The findings of this study demonstrate chest pain management variations across different patient racial affiliation subgroups, even when controlling for similar HEART scores. Further research focusing on the many possible confounding factors (e.g., previous stress test results, provider care patterns, patient attitudes) that may influence provider and patient decision-making processes is warranted to generate a fuller understanding of this cardiac care phenomenon.

### Conflict of Interest

None
